# Low 25(OH) D serum levels are related with hip fracture in postmenopausal women: a matched case–control study

**DOI:** 10.1186/s12967-015-0756-x

**Published:** 2015-12-23

**Authors:** Xing-Mao Fu, Shao-Guang Fan, Shu-Liang Li, Yi-Sheng Chen, Hai Wu, Yan-Long Guo

**Affiliations:** Institute of Traumatic Orthopedics, The 89th Hospital of People’s Liberation Army, No. 256 Beigongxi Str, Weifang, 261021 Shandong Province People’s Republic of China

**Keywords:** 25-Hydroxyvitamin D, Intact parathyroid hormone, Hip fracture, Upper limb fracture, Postmenopausal women

## Abstract

**Purpose:**

There is limited information on the prevalence of vitamin D deficiency among patients diagnosed with hip fracture in the Chinese Han population. Therefore, the aim of this study was to assess the effects of change in the serum levels of 25-hydroxyvitamin D [25(OH) D] and intact parathyroid hormone (iPTH) among postmenopausal women in North China with confirmed hip fracture.

**Methods:**

This study was done from May 1, 2012 to April 30, 2014. Three hundred and forty-nine postmenopausal women who were diagnosed with first-ever hip fracture and 349 matched controls without fracture were used for this study. The 25(OH) D, iPTH, alkaline phosphatase, calcium, and phosphorus levels were measured in fasting venous blood samples collected from the subjects. A predesigned questionnaire was used to collect information on covariates for multivariate analyses to evaluate the hypothesized relationship between vitamin D deficiency and fracture risk.

**Results:**

The serum 25(OH) D levels were found to be significantly (*P* < 0.0001) lower in hip fracture patients than in the controls [37.0 (interquartile range [IQR] 28.0–48.0) nmol/L vs. 41.3 (IQR 32.0–54.5) nmol/L; *P* < 0.0001], and the iPTH levels were significantly higher in the former group [10.2 (IQR 6.3–14.9) pmol/L vs. 5.8 (IQR 4.1–6.6) pmol/L; *P* < 0.0001]. Further, a 25(OH) D level ≤50 nmol/L was found to independently indicate the occurrence of hip fracture [odds ratio (OR), 3.023; 95 % confidence interval (CI) 2.154–4.298], as well as hip fracture with concomitant upper limb fracture (OR 4.473; 95 % CI 2.984–10.532). Similarly, a serum iPTH level ≥6.8 pmol/L independently indicated the development of hip fracture (OR 2.498; 95 % CI 1.764–3.942), as well as hip fracture with concomitant upper limb fracture (OR 3.254; 95 % CI 1.998–7.984).

**Conclusions:**

Vitamin D insufficiency and secondary hyperparathyroidism were found to be common problems in the sample of postmenopausal women who had experienced hip fracture. Monitoring the alterations in the serum levels of 25(OH) D and iPTH could be applied clinically as independent risk factors for hip fracture.

## Background

Vitamin D deficiency is a common medical condition worldwide. It usually develops because of insufficient sources of endogenous and exogenous vitamin D (inadequate intake or excessive consumption) and may eventually result in bone mass reduction, especially in elderly women [1]. The effect of inadequate vitamin D supply on bone health has been well established, as observed in the development of rickets in children and osteomalacia in adults [2]; both conditions that result from vitamin D deficiency. Age is a predominant risk factor for the development of hip fracture and the rapidly increasing number of elderly individuals worldwide highlights the urgency of implementing timely and suitable prevention strategies for patients with confirmed hip fracture.

A strong association between vitamin D deficiency and fracture development has been suggested, and thought to underlie the significant increases in mortality and morbidity rates of fracture patients [3, 4]. Even survivors of this condition have been reported to be at high risk of permanent disability. Rehabilitation results have not been optimistic, with almost a quarter of the patients unable to live independently and requiring long-term in-home supportive nursing care, and over three-fifths of these individuals unable to fully recover the same level of leg function prior to the fracture [5]. Vitamin D status is typically determined by measuring serum 25-hydroxyvitamin D [25(OH) D] levels. Piepe et al. [6] found that in elderly patients who had recently experienced hip fracture, vitamin D insufficiency was a commonly detected phenomenon and not a coincidental finding. Previous works have documented negative correlations between decreased serum levels of 25(OH) D and increased risk of hip fracture [7–9]. For example, in Japan, a recent study conducted at a general hospital found that about two-thirds of patients with acute hip fracture had vitamin D insufficiency [10]. In addition, previous studies have suggested that lower levels of 25(OH) D could be regarded as a risk marker for hip fracture [11, 12]. Given this correlation between low vitamin D level and increased risk for hip fracture, Lips et al. [13] reported that a daily vitamin D supplement of 800 IU in combination with calcium was an effective method to reduce the high incidence of non-vertebral fracture. The efficacy of vitamin D in preventing fractures could stem from its effects on muscle tissue (maintaining the normal structural and optimal functions of skeletal muscles), in addition to its known benefits in bone metabolism [14].

Hip fractures are estimated to account for a large proportion of osteoporosis cases, along with other severe consequences. A single fall is associated with both hip and upper limb fractures in about 4–5 % of older people who fracture their hip [15, 16]. Several reports have shown that decreased levels of 25(OH) D lead to the occurrence of hip and other fragility fractures, including those affecting the upper limbs; however, there are inconsistencies among the different studies [17]. In research conducted in India, Dhanwal et al. [18] found that hip fracture patients frequently had higher rates of vitamin D deficiency and secondary hyperparathyroidism. Unfortunately, no similar studies have been done in a sample of Chinese patients. Therefore, the aim of this preliminary study was to evaluate the serum 25(OH) D and intact parathyroid hormone (iPTH) levels in postmenopausal women diagnosed with hip fracture in the northern Chinese Han population.

## Subjects and methods

The study protocol was approved by the ethics committee of the 89th Hospital of People’s Liberation Army and was explained to all the participants included in the study. Written informed consent was obtained from eligible subjects in accordance with the Declaration of Helsinki.

From May 1, 2012 to April 30, 2014, participants were recruited at the Department of Orthopedics of the 89th Hospital of People’s Liberation Army (situated at 39°26′N latitude). The hospital is one of the best orthopedic hospitals in Shandong Province, China. Annually, the number of orthopedic outpatients is approximately 20,000 patients (the number seen in the hospital). In fact, the hospital receives a particular group of patients with complications. The inclusion criterion was predefined as postmenopausal women with confirmed first-ever hip fracture. The exclusion criteria included: (1) patients diagnosed with a fracture after a high-energy trauma, such as vehicular accidents, major falls, and direct injuries, among others; (2) patients with confirmed secondary tumor or primary hyperparathyroidism; (3) patients with other metabolic disorders or internal disease, such as hyperthyroidism, malignant tumor, chronic kidney disease, as well as autoimmune diseases; (4) patients taking calcium and/or vitamin D supplements. Among the patients with fractures were a subgroup consisting of subjects who sustained a concomitant fracture of the upper limb. In this subgroup, a single fall resulted in both a hip and an upper limb fracture. All the fractures were confirmed by X-ray examination, and all X-rays examinations were done by the same radiologist.

Postmenopausal women absent of hip fracture matched in age (±1 year), ethnicity, body mass index (BMI ±1), and common chronic diseases (hypertension, diabetes mellitus, hyperlipoproteinemia, and cardiovascular disease) were included as a control group. The controls came from a community located around the hospital. It was a random population sample. All control subjects were physically active (at work or able to perform home duties) within 30 days of the date of fracture diagnosis in the patient. If more than one potential control met the criteria, the control whose date of serum collection was closest to that of the patient was selected. Women who took calcium and/or vitamin D supplements were excluded. The exclusion criteria for patients also applied to the controls. Subjects who previously received estrogen treatment or other bone-active therapies were excluded from both the experimental and control groups.

A standardized questionnaire was predesigned to explore the hypothesized relationship between risk factors for fractures and vitamin D deficiency. At admission, all women (patients and controls) were instructed to answer the standardized questionnaire, which collected information on age, BMI, current tobacco use, season at time of blood collection, sun exposure (“adequate” was defined as over 15–20 min daily for 5 days per week or >2 h per week) [19], history of a fall in the last year, hip fracture type (cervical or trochanteric), cognitive impairment, neurologic impairment, infections, time between fracture occurrence and blood collection, number of concomitant diseases, surgical procedure type (arthroplasty or internal fixation), and family history of fragility fracture. Cognitive impairment was based mainly on the evaluation of the Mini-Mental State Examination score (24/30) [20]. Cognitive impairment was predefined as a score of less than 24 [21]. After the completion of records and careful evaluation of the patients’ information, neurologic disease-related impairments were examined and confirmed by clinical examination.

On the morning (8:00 AM) after the day of admission, fasting blood samples were obtained from all women (patients and controls) for the determination of serum 25(OH) D and iPTH levels. Blood samples (5 mL in total) were drawn from the cubital vein of each subject with the use of vacuum collection tubes. The collected serum samples were centrifuged for 10 min, then aliquots of the samples were refrigerated immediately at −80 °C until use. All the tests were done under the supervision of a qualified biochemist. The serum levels of calcium, phosphorous, high-sensitivity C-reactive protein (Hs-CRP) and alkaline phosphatase (ALP) were estimated by standard procedure with the use of an Olympus AU2700 analyzer (Olympus, Tokyo, Japan). The serum 25(OH) D levels were measured within the calibration range (7.5–175 nmol/L) by applying the E601 modular analytics (Roche Diagnostics, Mannheim, Germany). The intra-assay and inter-assay coefficients of variation (CV) were 4.6–7.9 and 5.8–9.7 %, respectively. The level of 25(OH) D was used to classify the vitamin D status as: (1) vitamin D deficiency (<50 nmol/L) or (2) vitamin D insufficiency (50–75 nmol/L) [22]. The chemiluminescence immunoassay method was used to detect the specific serum levels of iPTH with the use of the DPC Immulite 2000 system (Diagnostic Products Corporation, Los Angeles, CA, USA) within a calibration range of 0.3–263 pmol/L; the sensitivity of the method was 0.3 pmol/L. The intra-assay CV ranged from 2.88 to 3.93 %, whereas the inter-assay CV ranged from 3.89 to 5.02 %. The normal range based on laboratory standards is 1.3–6.8 pmol/L.

In this study, discrete variables are expressed as proportions or frequencies; these were compared by using the Chi square test between groups. Continuous variables are expressed as medians with an interquartile range (IQR); between-groups comparisons were done with the Mann–Whitney test or Student’s paired t test. Besides, Spearman’s test was applied to analyze specific correlations among continuous variables. Individual clinical information (age, BMI, current tobacco use, season of blood collection, sun exposure, history of a fall in the last year, hip fracture type, cognitive impairment, neurologic impairment, infections, time between fracture occurrence and blood collection, number of concomitant diseases, surgical procedure type and family history of fragility fracture) and other blood biomarkers (calcium, phosphorous, Hs-CRP and ALP) collected previously were measured as binary variables. Conditional logistic regression analysis was applied to determine the association between risk of fracture and alterations in serum 25(OH) D levels, after adjustment for possible confounders, namely, age, inadequate sun exposure, history of falls during the last year, neurologic impairment, cognitive impairment, and serum levels of Hs-CRP, ALP and iPTH. Experimental results on the relationship between changes in serum levels of 25(OH) D and the occurrence of fractures are expressed as adjusted odds ratios (OR) and 95 % confidence intervals (CI). Further, the receiver operating characteristic (ROC) curves were used in the evaluation of the accuracy of serum 25(OH) D levels in predicting fracture occurrences. The area under the ROC curve (AUC) was calculated as a measure of the accuracy of the test. Statistical significance was set at *P* < 0.05. All statistical analyses were done with the use of SPSS for Windows, version 20.0 (SPSS Inc., Chicago, IL, USA).

## Results

### Patient characteristics

From the initial 424 screened patients, 349 postmenopausal women were included in the study. Thirty-three cases diagnosed with hip fracture were excluded due to a major trauma or cancer affecting the bone, which contributed to the fractures; another 42 cases were excluded because of death or halfway transferring to other hospital. The median age of the sample was 64 years (IQR 55–74 years). The median time between fracture onset and blood collection was 17.9 h (IQR 11.2–14.6 h). None of the patients received treatment for osteoporosis before the fracture. One hundred and twenty-nine patients had associated comorbid illnesses, 88 had two or more comorbid illnesses, and the rest had a single comorbid illness (24.6 % of the patients with hypertension, 16.6 % with diabetes, 21.8 % with hyperlipoproteinemia and 12.9 % with cardiovascular disease). Among the patients, 16.0 % had a previous family history of fragility fractures, and 28.7 % reported receiving treatment for comorbid conditions [14.3 % of the patients received glucose lowering agent (4.3 % with thiazolidinedione, while 6.3 % with insulin), 22.6 % received antihypertensive drugs and 17.2 % used of lipid-lowering medication]. Among the 349 eligible patients in our study, 71.3 % of those with hip fracture were confirmed to have vitamin D deficiency, whereas 63.6 % was found to have a tendency for secondary hyperparathyroidism. Moreover, the incidences in the controls were 45.8 and 35.8 %, respectively. Table 1 presents the summary statistics for the subjects included in the study.

**Table 1:** Baseline characteristics of patients with hip fracture and controls

Parameters	Patients (n = 349)	Controls (n = 349)	*P*
Age (years), median (IQR)	64 (55–74)	64 (55–74)	NS
BMI (kg/m^2^, IQR)	25.7 (23.5–27.4)	25.8 (23.4–27.5)	NS
Smoker (%)	22.9	21.5	NS
Inadequate sun exposure (%)	53.0	30.9	<0.001
Season of blood collection (winter, %)	25.5	25.8	NS
History of falls during the last year, median (IQR)	3 (2–4)	1 (0–2)	0.018
Hip fracture type (%)		–	
Femoral head fracture	47.3		
Femoral neck fracture	44.4		
Subtrochanteric	8.3		
Concomitant upper-limb fractures (%)	8.3	–	
Distal radius (%)	5.7	–	
Proximal humerus (%)	2.6	–	
Patients had associated comorbid illnesses (%)	37.0	37.0	NS
Diabetes	16.6	16.3	NS
Hypertension	24.6	25.2	NS
Cardiovascular disease	12.9	12.6	NS
Hyperlipoproteinemia	21.8	21.8	NS
Receiving treatment for comorbid conditions (%)	28.7	28.4	NS
Glucose treatment	14.3	13.8	NS
Blood pressure treatment	22.6	21.5	NS
Use of lipid-lowering medication	17.2	16.3	NS
MMSE < 24 (%)	22.1	10.0	0.025
Neurologic impairment (%)	18.9	8.3	0.027
Time between fracture and blood collection (hs) median (IQR)	17.9 (11.2–24.6)	–	
Hospital stay (days), median (IQR)	13 (8–19)	–	
Laboratory findings (IQR)			
Serum calcium (mmol/L)	2.39 (2.25–2.47)	2.48 (2.32–2.62)	0.032
Serum phosphate (mmol/L)	1.49 (1.13–1.76)	1.51 (1.20–1.77)	NS
Serum ALP (IU/L)	246 (145–342)	159 (100–236)	<0.001
Serum Hs-CRP (mg dL^−1^)	0.36 (0.22–0.42)	0.29 (0.18–0.36)	0.002
Serum iPTH (pmol/L)	10.2 (6.3–14.9)	5.8 (4.1–6.6)	<0.0001
Serum 25(OH) D (nmol/L)	37.0 (28.0–48.0)	41.3 (32.0–54.5)	<0.0001
Vitamin D deficiency [n (%)]	71.3	45.8	<0.0001
Hyperparathyroidism [n (%)]	63.6	35.8	<0.0001

Results are expressed as percentages or as medians (IQR)

*NS* no significant, *BMI* body mass index, *MMSE* Mini-Mental State Examination score, *Hs*-*CRP* high-sensitivity-C-reactive protein (BMI), *ALP* alkaline phosphatase, *iPTH* intact parathyroid hormone

### Main results

**Fig. 1:**
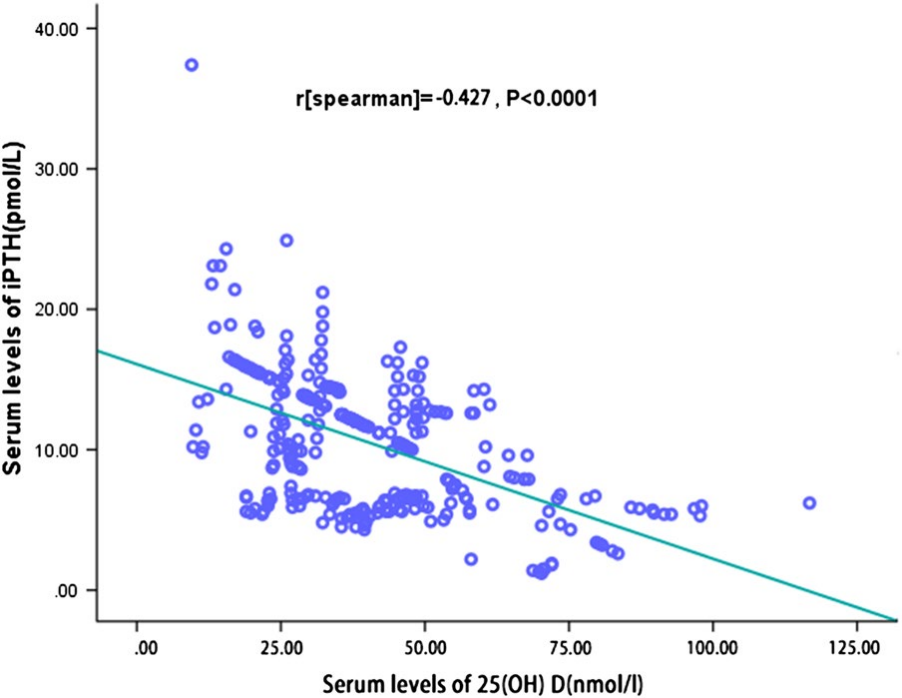
Correlation between the serum 25(OH) D levels and iPTH in postmenopausal women with hip fractures (n = 349). *iPTH* intact parathyroid hormone

The serum 25(OH) D levels were found to be significantly (*P* < 0.0001) lower in hip fracture patients than in the controls, with an apparent statistical difference [37.0 (IQR 28.0–48.0) nmol/L vs. 41.3 (IQR 32.0–54.5) nmol/L; *P* < 0.0001]. Furthermore, there was an obvious difference in serum iPTH levels between groups, with significantly higher levels in patients than in the controls [10.2 (IQR 6.3–14.9) pmol/L vs. 5.8 (IQR 4.1–6.6) pmol/L; *P* < 0.0001]. As shown in Fig. 1, the Spearman’s test results indicated a negative relationship between the serum levels of 25(OH) D and iPTH in hip fracture patients (r = −0.427, *P* < 0.0001). Similarly, there was also a negative relationship between the serum levels of 25(OH) D and iPTH in controls (r = −0.301, *P* < 0.0001). In addition, there were significant, although weak, correlations between the 25(OH) D levels and those of high-sensitivity C-reactive protein (r = −0.162, *P* = 0.002) and Ca (r = 0.202, *P* < 0.0001) in hip fracture patients. Interestingly, there was a negative relationship between the serum levels of 25(OH) D and the number of falls in hip fracture patients (r = −0.532, *P* < 0.0001), while a similarly negative relationship was found in controls (r = −0.356, *P* < 0.0001). Blood samples were taken over the course of the four seasons and the serum levels of 25(OH) D were compared between groups. Analysis of variance revealed significant seasonal differences in 25(OH) D levels (*P* = 0.003). The patients who entered the study during the winter had lower serum levels of 25(OH) D than the patients who entered the study during the other three seasons [35.4 (IQR 25.7–44.6) nmol/L vs. 38.2 (IQR 29.2–49.6) nmol/L; *P* < 0.0001]. No correlations were found between the serum levels of 25(OH) D and the remaining binary variables (all, *P* > 0.05).

### 25(OH) D and risk of hip fracture

Based on the ROC curve for the screening of hip fracture risks, the optimal cutoff level of serum 25(OH) D was estimated to be 48.0 nmol/L, which yielded a sensitivity of 70.6 % and a specificity of 72.3 % (AUC 0.701; 95 % CI 0.624–0.779). Table 2 shows that after adjustment for possible confounders, the multivariate conditional logistic regression analysis results suggested an increased risk of hip fracture when the serum 25(OH) D level was ≤50 nmol/L (OR 3.023; 95 % CI 2.154–4.298; *P* < 0.001) and the serum iPTH level was ≥6.8 pmol/L (OR 2.498; 95 % CI 1.764–3.942; *P* < 0.001).

**Table 2:** Univariate and multivariate conditional logistic regression analysis for hip and upper-limb fracture

Parameter	Univariate analysis			Multivariate analysis		
	OR^a^	95 % CI^a^	*P*	OR^a^	95 % CI^a^	*P*
Predictor: hip fracture (N = 349)						
25(OH) D (≤50 nmol/L)	3.779	1.346–7.754	<0.0001	3.023	2.154–4.298	<0.0001
Age	1.224	1.057–1.765	0.007	1.187	1.095–1.432	0.011
Inadequate sun exposure	1.154	1.056–1.554	0.028	1.067	1.015–1.465	0.042
History of falls during the last year	1.998	1.213–2.876	0.026	1.675	0.954–3.986	0.167
Neurologic impairment	3.332	1.487–5.432	0.035	2.443	0.713–4.876	0.675
Cognitive impairment	1.453	1.109–1.765	0.022	1.287	0.995–3.054	0.265
Hs-CRP	1.119	1.032–1.332	<0.001	1.075	1.024–1.242	0.001
ALP	1.342	1.114–1.567	<0.001	1.211	1.068–1.243	0.009
iPTH (≥6.8 pmol/L)	3.223	1.635–6.754	<0.0001	2.498	1.764–3.942	<0.001
Predictor: hip and upper-limb fracture (N = 29)						
25(OH) D (≤50 nmol/L)	6.985	1.152–21.763	<0.0001	4.473	2.984–10.532	<0.0001
Age	1.197	1.047–1.455	<0.001	1.088	1.027–1.133	<0.001
BMI	1.332	1.142–1.487	0.006	1.225	1.087–1.392	0.009
Inadequate sun exposure	1.176	1.032–1.543	0.010	1.082	1.012–1.544	0.022
History of falls during the last year	2.221	1.317–2.678	0.012	1.785	0.932–3.576	0.113
Neurologic impairment	2.543	1.943–4.581	0.036	1.476	0.911–3.632	0.455
Cognitive impairment	1.665	1.232–2.043	0.020	1.332	0.992–2.874	0.321
Hs-CRP	1.228	1.053–1.489	0.005	1.112	1.044–1.432	0.015
ALP	1.186	1.105–1.325	0.003	1.142	1.054–1.375	0.013
iPTH (≥6.8 pmol/L)	4.214	2.536–8.433	<0.0001	3.254	1.998–7.984	<0.001

*OR* odds ratio, *CI* confidence interval, *BMI* body mass index, *Hs*-*CRP* high-sensitivity-C-reactive protein, *ALP* alkaline phosphatase, *iPTH* intact parathyroid hormone

^a^Note that the odds ratio corresponds to a unit increase in the explanatory variable

### 25(OH) D and risk of hip and upper limb fracture

In this study, 29 hip fracture patients had concomitant upper limb fracture. The subgroup analysis results indicated that the serum 25(OH) D levels were significantly (*P* < 0.0001) higher in patients with hip fracture only compared with those who also had upper limb fracture [37.5 (IQR 29.0–48.5) nmol/L vs. 26.8 (IQR 17.0–39.5) nmol/L]. Among hip fracture patients who also had upper limb fracture, 85.9 and 68.8 % had vitamin D deficiency and secondary hyperparathyroidism, respectively.

Furthermore, the optimal cutoff level of serum 25(OH) D as an indicator for the screening of hip and upper limb fracture was estimated to be 43.8 nmol/L based on the observation of the ROC curve; this yielded a sensitivity of 73.8 % and a specificity of 67.4 %, with the AUC at 0.724 (95 % CI 0.621–0.823). Again, with an AUC of 0.724, 25(OH) D showed a significantly greater discriminatory ability compared with iPTH (AUC 0.647; 95 % CI 0.564–0.743; *P* < 0.01), high-sensitivity C-reactive protein (AUC 0.672; 95 % CI 0.557–0.785; *P* < 0.01), and age (AUC 0.623; 95 % CI 0.545–0.737; *P* < 0.001). Table 2 shows the ORs of the 25(OH) D and iPTH levels compared with those of other clinical risk factors, as obtained through univariate conditional logistic regression analysis. Similarly, when the measured serum 25(OH) D level was ≤50 nmol/L (OR 4.473; 95 % CI 2.984–10.532; *P* < 0.0001), the multivariate conditional logistic regression analysis showed a possibly increased risk of upper limb fracture. When the measured serum iPTH level was ≥6.8 pmol/L (OR 3.254; 95 % CI 1.998–7.984; *P* < 0.001), an increased risk of hip fracture was also found.

## Discussion

This study investigated the correlation between serum levels of 25(OH) D and iPTH, and accuracy in indicating the risk of hip and upper limb fracture in postmenopausal women in a northern Chinese Han sample. Several reports have hypothesized a possible association between 25(OH) D levels and the occurrence of hip fracture. However, the biggest advantage of this study was that it focused on the relationship of these two biomarkers with the occurrence of hip and upper limb fracture in a sample of ethnic northern Chinese patients. Few studies have linked the deficiency of such biomarkers to the occurrence of hip and upper limb fracture, and none have been done in a Chinese sample thus far.

The main finding in our study was that low serum 25(OH) D and high iPTH levels indicate a higher risk of hip and upper limb fracture in the northern Chinese population. Serum 25(OH) D levels ≤50 nmol/L was associated with a 4.473-fold increase in risk of concomitant upper limb fracture in postmenopausal women with hip fracture. Therefore, postmenopausal women with insufficient vitamin D may be at higher risk of sustaining fractures. Consistent with our results, Oyen et al. [23] reported that vitamin D status had a significant inverse association with risk of low-energy distal radius fracture in both women and men. In addition, Cauley et al. [24] found a negative correlation between decreased levels of serum 25(OH) D and higher risk of hip fracture. Similarly, Holvik et al. [25] reported that subjects with a lower quartile level of serum 25(OH) D tended to have an increased risk of hip fracture. Burgi et al. [26] found that among white women, those with serum 25(OH) D levels in the highest quartile had half the risk of stress fracture compared with those in the lowest quartile (*P* < 0.05). Interestingly, our findings support those results. However, contrasting findings have also been previously reported, with some authors arguing that no such significant association exists after evaluating the possible incidence of vertebral fracture [27]. Van et al. [28] reported that a serum level of 25(OH) D <30 nmol/L was associated with an increased risk of fractures in the youngest, but not in the oldest, age group after adjustment for confounders. Interestingly, Liu et al. [29] found that there was an association between serum 25(OH) D levels and prognosis in Chinese postmenopausal women with hip fracture. Differences in the assay and sample test methods, subject populations, and analytic platforms used may explain the inconsistencies among studies. However, more research is necessary to clarify the difference.

In the United States, for example, more than half of women with serum vitamin D levels <30 nmol/L were found to have hip osteoporotic fracture [30]. Similarly, 21.6 % of patients with hip fracture in Italy were found to have hypovitaminosis D [31]. In a study in Japan, Sakuma et al. [10] reported that 62 % of patients with hip fracture had hypovitaminosis D, whereas 19.4 % had secondary hyperparathyroidism (serum iPTH level >65 pg/mL). In Spain, 67 % of subjects with hip fracture had vitamin D levels less than 50 nmol/L, and 55 % had increased iPTH [32]. Furthermore, over 76.7 and 68.9 % of patients with hip fracture in India had vitamin D deficiency and secondary hyperparathyroidism, respectively [18]. In addition, Dhanwal et al. [18] observed that there was a significant inverse correlation of iPTH level with 25(OH) D in patients with hip fracture. In contrast, an earlier study found no such correlation [33]. In our study, 72.3 % of subjects with hip fracture had vitamin D levels less than 50 nmol/L, and 63.6 % had hyperparathyroidism. Similarly, in another Chinese sample, almost four-fifths of the hip fracture patients (78.9 %) had vitamin D deficiency [29].

Severe vitamin D deficiency may be involved in the genesis of concomitant fractures. However, the biological mechanism linking vitamin D deficiency with fracture is still unclear. Some biologically plausible mechanisms could be largely responsible for the risk of fracture. First, vitamin D deficiency may exert an effect on the reduction of muscle mass, which in turn may be the possible reason for the occurrence of hip fractures [34]. To be specific, vitamin D may play a part in maintaining the normal structural and optimal functions of skeletal muscles, whereas vitamin D deficiency inevitably leads to loss of muscle mass and weakening of muscle function [5]. Besides, vitamin D deficiency may be closely associated with the production of abnormal levels of calcium-phosphorus, consequently resulting in diminished collagen matrix mineralization [35]. Jesudason et al. [36] found that, in postmenopausal women, when the serum 25(OH) level <60 nmol/L, there might be an increase considering some bone resorption markers and ALP. By coincidence, the present analysis showed a negative correlation between 25(OH) D and ALP. Second, another possible mechanism might be that vitamin D deficiency affects bone cells that express the vitamin D receptors (VDR), which has a possible effect on modulating the process of bone turnover [34]. In addition, similarly to another study, vitamin D deficiency has been found to contribute to the increased rate of falls, indicating a significant relationship between falls and VDR gene polymorphisms [37]. Further, in the experiment of McClung et al. [38], two polymorphisms (Fok1 and Bsm1) in the VDR were observed to be significantly correlated with increased risk of stress fracture. Third, the results of our study suggested that secondary hyperparathyroidism in vitamin D-depleted subjects may have a large influence on the incidence of impaired muscle function [14]. Lopes et al. [39] found that vitamin D deficiency-induced secondary hyperparathyroidism increased bone turnover and loss and therefore promoted the occurrence of fracture. Lastly, previous evidence has shown the existence of a significant relationship between vitamin D status and both fall pattern and choice reaction time. By definition, choice reaction time is a measurement that is especially useful in the reflection of neuroprotective mechanisms (e.g., central processing, cognition, motor response, etc.) [17, 34]. Previous works have supported the idea that by affecting bone tissue, severe vitamin D deficiency may increase the risk of multiple concomitant fractures [40].

This study has several limitations that should be taken into consideration. First, we were not able to examine other risk factors for fracture, including daily diet, physical activity, and physical performance. Vitamin D can improve the muscle function. Unfortunately, we did not test muscle function in this study. Second, we did not obtain data on the pre-fracture levels of serum 25(OH) D. In addition, we also did not obtain data on the dietary calcium and vitamin D intake, which might affect the levels of 25(OH) D, ALP and iPTH in the serum [41]. Further study about dietary calcium and vitamin D intake should be registered. Third, data collection toward further estimation of bone fragility in our patients, such as measurement of bone mineral density and determination of the prevalence of vertebral fracture, was not carried out. Fourth, the serum 25(OH) D concentrations in male patients were not measured. Therefore, this study could not determine whether a similar association between lower serum 25(OH) D concentration and higher risk of stress fracture as that found in females exists in males; a previous study done by Ruohola et al. [42] in Finland identified such an association in males. Fifth, we did not have the resources to measure the serum vitamin D–binding protein in our samples. Vitamin D–binding protein may play role in the process of fracture constant. Future studies with data on vitamin D–binding protein and vitamin D status are needed to further clarify the effect of each on fracture risk. Finally, the number of study subjects was small and not replicated. Hip fracture cases in our study perhaps do not represent all female hip fractures in Shandong because this hospital often treats complicated cases. Larger more extensive studies are needed to evaluate the risk factors of fracture in Chinese subjects to determine whether serum 25(OH) D is a reliable marker of fractures that can be used in clinical practice in other samples.

## Conclusions

Vitamin D insufficiency and secondary hyperparathyroidism were found to be common problems in the sample of postmenopausal women who had experienced hip fracture. Monitoring the alterations in the serum levels of 25(OH) D and iPTH could be applied clinically as independent risk factors for hip fracture.

## Abbreviations

25(OH) D: 25-hydroxyvitamin D
iPTH: intact parathyroid hormone
BMI: body mass index
MMSE: Mini-Mental State Examination score
Hs-CRP: high-sensitivity C-reactive protein
ALP: alkaline phosphatase
CV: coefficients of variation
IQR: interquartile range
OR: odds ratios
CI: confidence intervals
ROC: receiver operating characteristic
AUC: area under the ROC curve
VDR: vitamin D receptors
